# Mechanism of active component β-sitosterol from Myristica fragrans inducing apoptosis in bladder cancer cells via regulating the BCL-2/BAX/caspase-3 pathway

**DOI:** 10.3389/fonc.2025.1698721

**Published:** 2025-11-03

**Authors:** Yongkang Zhu, Zhao Tang, Zhaoyue Lu, Dongyang Gao, Hao Pan, Haozhe Jiang, Zhen Zhang, Huiqing Zhang

**Affiliations:** ^1^ Department of Urology (Ward I), The First Affiliated Hospital of Xinxiang Medical University, Xinxiang, Henan, China; ^2^ Life Science Center, The First Affiliated Hospital of Xinxiang Medical University, Xinxiang, Henan, China; ^3^ Xinxiang Municipal Key Laboratory for Diagnosis and Treatment of Lower Urinary Tract Obstruction, Xinxiang, Henan, China; ^4^ Henan Provincial Key Laboratory of Urodynamics and Pelvic Floor Reconstruction Medicine, Xinxiang, Henan, China

**Keywords:** Myristica fragrans, bladder cancer, network pharmacology, β-sitosterol, apoptosis

## Abstract

**Background:**

Nutmeg (Myristica fragrans) has been traditionally used in herbal medicine, but its potential anti-cancer effects remain largely unexplored. This study aimed to investigate the molecular mechanisms of nutmeg against bladder cancer through an integrated strategy combining network pharmacology, molecular docking, and *in vitro* validation.

**Methods:**

Active compounds of nutmeg were retrieved from the TCMSP and PubChem databases using oral bioavailability (OB ≥30%) and drug-likeness (DL ≥0.18) as criteria. Potential targets were predicted using SwissTargetPrediction and cross-referenced with bladder cancer-related genes from GeneCards, OMIM, and TTD. Common targets were analyzed by STRING, Cytoscape, and DAVID for PPI, GO, and KEGG enrichment. Molecular docking was performed to evaluate binding affinities between candidate compounds and core targets. *In vitro* experiments, including CCK-8, colony formation, wound-healing, Transwell, flow cytometry, and Western blotting, were conducted to validate the anti-tumor effects of β-sitosterol on T24 and 5637 bladder cancer cells.

**Results:**

Nine active compounds were identified, with β-sitosterol emerging as the key candidate. A total of 284 overlapping targets were obtained between nutmeg and bladder cancer. GO and KEGG enrichment suggested significant involvement in apoptosis and PI3K-Akt signaling pathways. Molecular docking showed that β-sitosterol exhibited strong binding to BCL-2 (–8.6 kcal/mol) and CASP3 (–8.3 kcal/mol). *In vitro*, β-sitosterol significantly reduced cell viability (IC_50_: 50 μM for 5637, 60 μM for T24), inhibited proliferation, colony formation, and migration, and induced apoptosis in a dose-dependent manner. Western blot confirmed upregulation of Bax and cleaved Caspase-3 and downregulation of BCL-2.

**Conclusion:**

This study demonstrates that β-sitosterol, a major bioactive compound of nutmeg, suppresses bladder cancer progression by modulating the BCL-2/Bax/Caspase-3 axis and PI3K-Akt signaling pathway. These findings provide novel insights into the therapeutic potential of nutmeg as a complementary strategy for bladder cancer treatment.

## 1 Introduction

Bladder cancer is the tenth most common malignancy worldwide and represents the most prevalent cancer of the urinary system. Its incidence shows a marked gender disparity, with men being approximately three to four times more frequently affected than women (1). According to recent global statistics, about 573,000 new cases and 213,000 related deaths occur annually. Major risk factors include cigarette smoking, occupational exposure to aromatic amines, chronic inflammation of the bladder, and inherited susceptibility (2, 3). Histopathologically, bladder cancer is categorized into non-muscle-invasive (NMIBC) and muscle-invasive (MIBC) types. NMIBC constitutes roughly 75% of initial diagnoses and, despite generally favorable early outcomes, exhibits a recurrence rate of 50–70% (4). In contrast, MIBC is characterized by aggressive invasion and metastasis, with a five-year survival rate below 50% (5). Recent studies have revealed the biological complexity of bladder cancer. Crosstalk between the nervous system and tumor microenvironment has been implicated in tumor progression and resistance to therapy (6). Patient-derived xenograft models have further highlighted their profound heterogeneity and translational challenges (7). Moreover, exosome-mediated signaling has been shown to contribute to chemoresistance mechanisms (8). Current standard management relies on surgery, chemotherapy, radiotherapy, and immunotherapy. However, conventional chemotherapeutic agents such as cisplatin and gemcitabine often cause severe systemic toxicity and readily induce resistance (9). Immune checkpoint inhibitors targeting PD-1/PD-L1 can benefit certain patients, yet their overall response rates remain limited (10). These limitations underscore the pressing need for novel therapeutic strategies. Among emerging approaches, the systematic exploration of natural bioactive compounds offers a promising direction for overcoming existing treatment bottlenecks, providing safer and multi-targeted alternatives.

Natural products and bioactive compounds derived from traditional Chinese medicine (TCM) have received growing attention in cancer research. Their multi-target actions, low toxicity, and potential synergistic effects make them promising candidates for novel anticancer drug development (11). Numerous studies have demonstrated that TCM formulations can inhibit tumor growth, induce apoptosis, and suppress metastasis through the coordinated regulation of multiple molecular pathways (12). Network pharmacology, integrating principles from chemistry, biology, and pharmacology, provides a powerful approach to uncover the complex relationships among bioactive compounds, molecular targets, and signaling networks in TCM (9, 13). This systems-level strategy helps elucidate the multi-component and multi-target nature of TCM and supports its modernization through evidence-based research.

Nutmeg (*Myristica fragrans*) is a widely used medicinal and dietary herb with reported anti-inflammatory, antioxidant, and antitumor properties. Among its bioactive constituents, β-sitosterol has been extensively studied and shown to suppress tumor progression in several cancer types, including breast, prostate, and lung cancers (10, 11, 14). However, the potential role of nutmeg-derived β-sitosterol in bladder cancer remains largely unexplored. Unlike prior studies that investigated β-sitosterol in isolation, our work establishes a direct mechanistic link between nutmeg-derived β-sitosterol and apoptosis induction in bladder cancer cells. Moreover, we explore whether nutmeg’s multi-component composition may enhance the pharmacological relevance of β-sitosterol in this context.

Therefore, this study integrates network pharmacology, molecular docking, and *in vitro* validation to elucidate the molecular mechanisms by which nutmeg-derived β-sitosterol suppresses bladder cancer cell growth and induces apoptosis. By focusing on apoptosis-related signaling and complementary pathways, this work provides novel insights into the therapeutic potential of β-sitosterol and nutmeg in bladder cancer treatment.

## 2 Materials and methods

### 2.1 Cell lines and culture

Human bladder cancer cell lines T24 and 5637 were obtained from the Cell Bank of the Chinese Academy of Sciences (Shanghai, China). Cells were cultured in RPMI-1640 medium supplemented with 10% fetal bovine serum (FBS) and maintained under standard conditions (37 °C, 5% CO_2_, humidified atmosphere). No human participants or animals were involved in this study; therefore, ethical approval was not required.

### 2.2 Reagents and antibodies

β-Sitosterol (purity ≥95%, CAS: 83-46-5) was purchased from Yuanye Biotechnology (Shanghai, China). RPMI-1640 medium and FBS were obtained from Zhongqiao Xinzhou Biotechnology (Shanghai, China). Cell Counting Kit-8 (CCK-8) was purchased from Sicojet Biotechnology (Shandong, China). Antibodies against BCL-2, Bax, Caspase-3, and GAPDH were obtained from Proteintech (Wuhan, China).

### 2.3 Network pharmacology analysis

#### 2.3.1 Identification of active compounds and targets

Nutmeg compounds were retrieved from the Traditional Chinese Medicine Systems Pharmacology (TCMSP) database (https://www.tcmsp-e.com/) using oral bioavailability (OB) ≥30% and drug-likeness (DL) ≥0.18 as screening criteria. Chemical structures and SMILES formats were confirmed using PubChem (https://pubchem.ncbi.nlm.nih.gov/). Potential targets were predicted by SwissTargetPrediction (restricted to *Homo sapiens*) and standardized using UniProt (https://www.uniprot.org/).

#### 2.3.2 Collection of bladder cancer targets

Bladder cancer-associated genes were obtained from GeneCards (https://www.genecards.org/), OMIM (https://www.omim.org/), and the Therapeutic Target Database (TTD, https://db.idrblab.net/ttd/). Duplicate targets were removed to construct the final disease-related target set.

#### 2.3.3 Identification of overlapping targets

Intersection of nutmeg- and bladder cancer-related targets was performed using an online Venn diagram tool (http://www.bioinformatics.com.cn/).

#### 2.3.4 Network construction and PPI analysis

The overlapping targets were uploaded to STRING (https://string-db.org/) with the organism restricted to *Homo sapiens* and the minimum required interaction score set at 0.4 for protein–protein interaction (PPI) network construction. The results were visualized using Cytoscape 3.10.0, and hub genes were identified using the cytoNCA plugin based on Degree, Betweenness, and Closeness parameters.

#### 2.3.5 GO and KEGG enrichment analysis

Gene Ontology (GO) and Kyoto Encyclopedia of Genes and Genomes (KEGG) enrichment analyses were performed using DAVID (https://davidbioinformatics.nih.gov/summary.jsp), with the species limited to *Homo sapiens* and significance thresholds set at *p* < 0.05 and FDR < 0.05. Results were visualized using the MicroBioinformatics platform (http://www.bioinformatics.com.cn/).

#### 2.3.6 Molecular docking

Crystal structures of target proteins were retrieved from the Protein Data Bank (PDB, https://www.rcsb.org/). Ligand structures were prepared using PubChem. Molecular docking was performed using CB-Dock2 (https://cadd.labshare.cn/cb-dock2/), and binding energies were calculated. Docking conformations were visualized using Discovery Studio 2021.

### 2.4 *In vitro* experiments

#### 2.4.1 Cell viability and IC_50_ determination

Cells were seeded in 96-well plates (1 × 10^4^ cells/well) and treated with β-sitosterol at various concentrations (0–90 μM) for 24 h. Viability was measured using the CCK-8 assay, and absorbance was recorded at 450 nm. IC_50_ values were calculated using GraphPad Prism 9.0.

#### 2.4.2 Cell proliferation assay

Cells were exposed to β-sitosterol for 24, 48, and 72 h. Cell proliferation was assessed using the CCK-8 assay as described above.

#### 2.4.3 Colony formation assay

T24 and 5637 cells (1,000 cells/well) were seeded in 6-well plates and treated with β-sitosterol. After 9 days, colonies were fixed with paraformaldehyde, stained with crystal violet, and counted (colonies ≥50 μm were considered viable).

#### 2.4.4 Migration assays

##### 2.4.4.1 Wound-healing assay

Confluent monolayers were scratched using a sterile pipette tip and cultured in medium containing 1% FBS with or without β-sitosterol. Images were captured at 0 and 24 h, and wound closure was quantified using ImageJ.

##### 2.4.4.2 Transwell migration assay

Cells (1.5 × 10^4^) were seeded into the upper chamber with serum-free medium. The lower chamber contained medium with 10% FBS. After 48 h, migrated cells were fixed, stained with crystal violet, and counted under a microscope.

#### 2.4.5 Apoptosis assay

Apoptosis was assessed by flow cytometry using Annexin V-FITC/PI double staining after 24 h treatment with β-sitosterol. Data were analyzed using FlowJo software.

#### 2.4.6 Western blotting

Cells were lysed in RIPA buffer supplemented with protease inhibitors, and protein concentrations were determined using a BCA assay. Equal amounts of protein (30–50 μg) were separated by SDS-PAGE and transferred to PVDF membranes. Membranes were blocked with 5% non-fat milk for 1 h at room temperature and incubated overnight at 4 °C with primary antibodies against BCL-2 (1:1000), Bax (1:1000), Caspase-3 (1:1000), and GAPDH (1:5000) (all from Proteintech, Wuhan, China). After washing, membranes were incubated with HRP-conjugated secondary antibodies (1:5000) for 1 h at room temperature. Protein bands were detected using enhanced chemiluminescence (ECL) and imaged with a Bio-Rad ChemiDoc system. Densitometric analysis was performed with ImageJ software, and protein expression levels were normalized to GAPDH. Data were obtained from at least three independent experiments and analyzed using one-way ANOVA, with *p* < 0.05 considered statistically significant.

### 2.5 Statistical analysis

Data are expressed as mean ± standard deviation (SD) from at least three independent experiments. Statistical analysis was performed using SPSS 31.0. Comparisons between groups were made using one-way ANOVA followed by least significant difference (LSD) tests. A P value <0.05 was considered statistically significant.

## 3 Results

### 3.1 Screening of active components in nutmeg

Based on the TCMSP and PubChem databases, a total of nine candidate compounds were identified from nutmeg (Myristica fragrans) using the criteria of oral bioavailability (OB ≥ 30%) and drug-likeness (DL ≥ 0.18). The screened compounds and their pharmacokinetic parameters are summarized in **Table 1**. Notably, β-sitosterol (OB = 36.91%, DL = 0.75) was highlighted as a potential core bioactive compound.

**Table 1:** Active components screened from Myristica fragrans.

Active component	MOL ID	Chinese name	Molecule name	OB	DL
RDK1	MOL000358	β-谷甾醇	beta-sitosterol	36.91	0.75
RDK2	MOL007920	2号化合物	meso-1,4-Bis-(4-hydroxy-3-methoxyphenyl)-2,3-dimethylbutane	31.31	0.26
RDK3	MOL009243	异瓜亚辛	Isoguaiacin	48.78	0.31
RDK4	MOL009254	加尔巴新	Galbacin	60.99	0.53
RDK5	MOL009255	5号化合物	5-[(2S,3S)-7-methoxy-3-methyl-5-[(E)-prop-1-enyl]-2,3-dihydrobenzofuran-2-yl]-1,3-benzodioxole	53.11	0.4
RDK6	MOL009259	氯菊脂	Kudos	45.05	0.37
RDK7	MOL009263	7号化合物	saucernetindiol	41.85	0.32
RDK8	MOL009264	8号化合物	Tetrahydrofuroguaiacin B	62.85	0.32
RDK9	MOL009265	9号化合物	threo-austrobailignan-5	49.48	0.31

### 3.2 Prediction of potential targets

**Figure 1:**
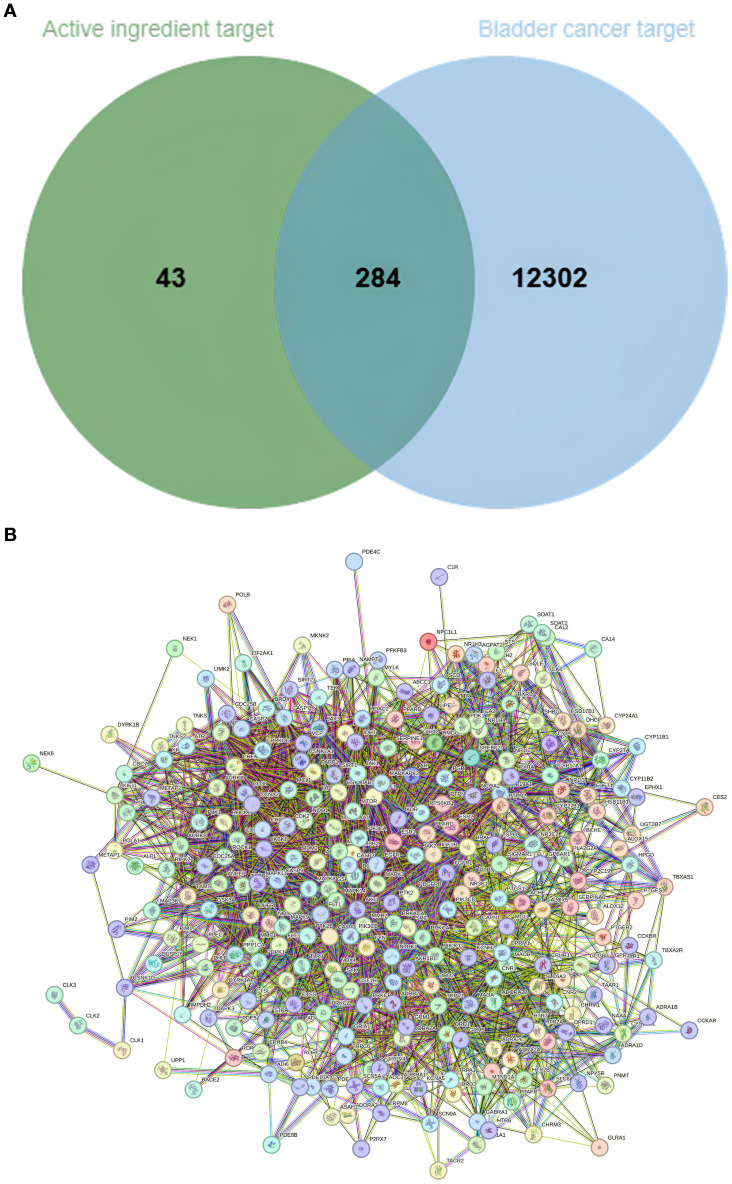
**(A)** Venn diagram of overlapping targets between nutmeg active components and bladder cancer. **(B)** PPI network of nutmeg active components and bladder cancer targets.

Potential targets of the nine candidate compounds were predicted using SwissTargetPrediction (Homo sapiens). After merging and deduplication, 327 unique targets were obtained. In parallel, bladder cancer-related targets were collected from the GeneCards, OMIM, and TTD databases, yielding 1,258 unique targets. Intersection analysis identified 284 common targets, representing potential anti-bladder cancer targets of nutmeg (**Figure 1A**). Protein-protein interaction (PPI) network construction further revealed several hub targets (**Figure 1B**).

### 3.3 Construction of component–target networks

**Figure 2:**
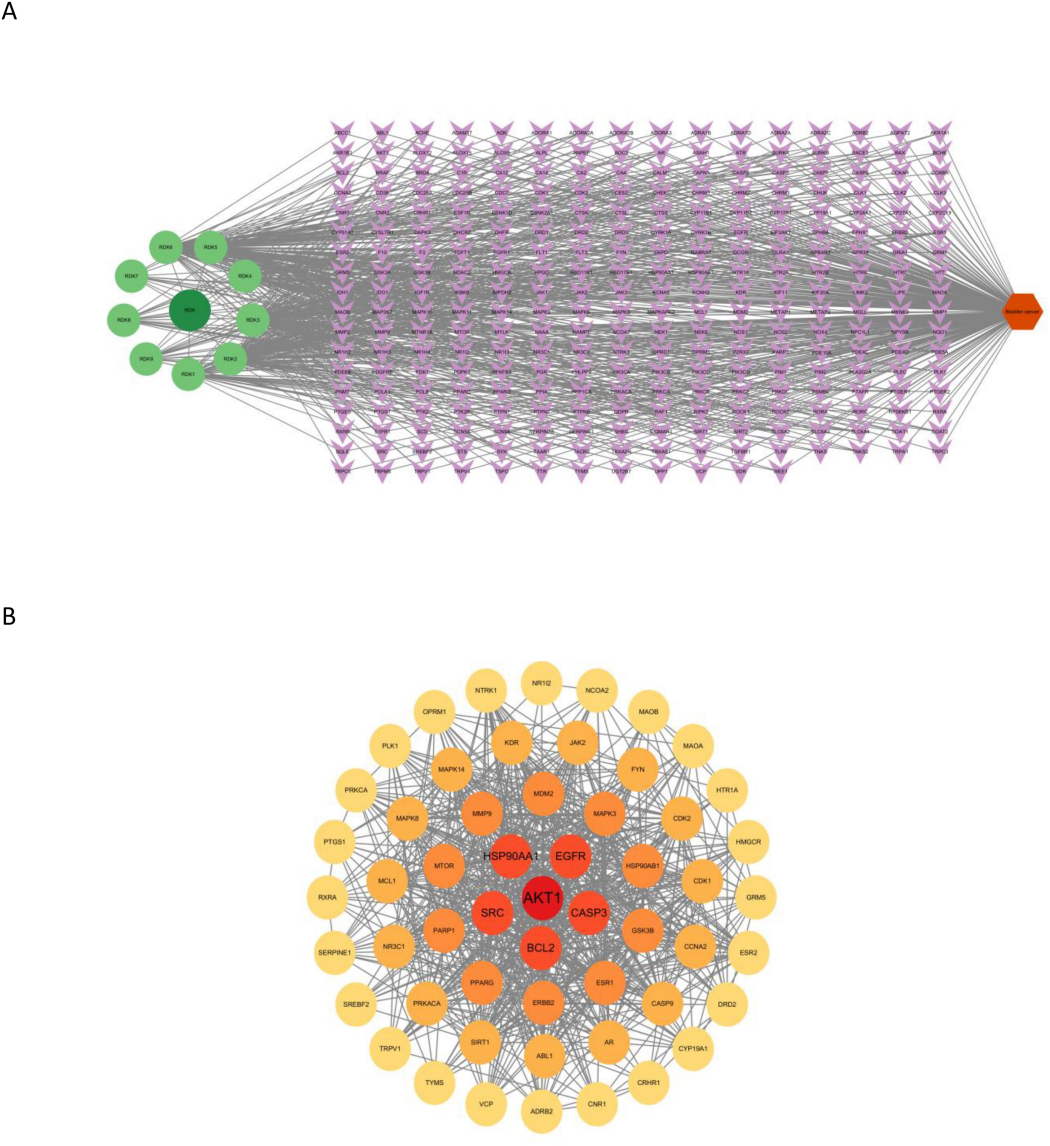
**(A)** Active component–target network of nutmeg against bladder cancer. In the figure, dark green circular nodes represent the drug, light green circular nodes represent active ingredients, purple diamond-shaped nodes represent target proteins, and orange hexagons represent the disease. **(B)** Compound–core target network of nutmeg. The circular nodes in the figure represent key core targets, with darker colors indicating higher Degree values.

To further elucidate the relationships between active compounds and potential targets, an active component–target network and a compound–core target subnetwork were constructed. These networks revealed that β-sitosterol and other major compounds exhibited strong associations with multiple cancer-related targets (**Figures 2A**, **B**).

### 2.4 GO Functional enrichment and KEGG pathway analysis

**Figure 3:**
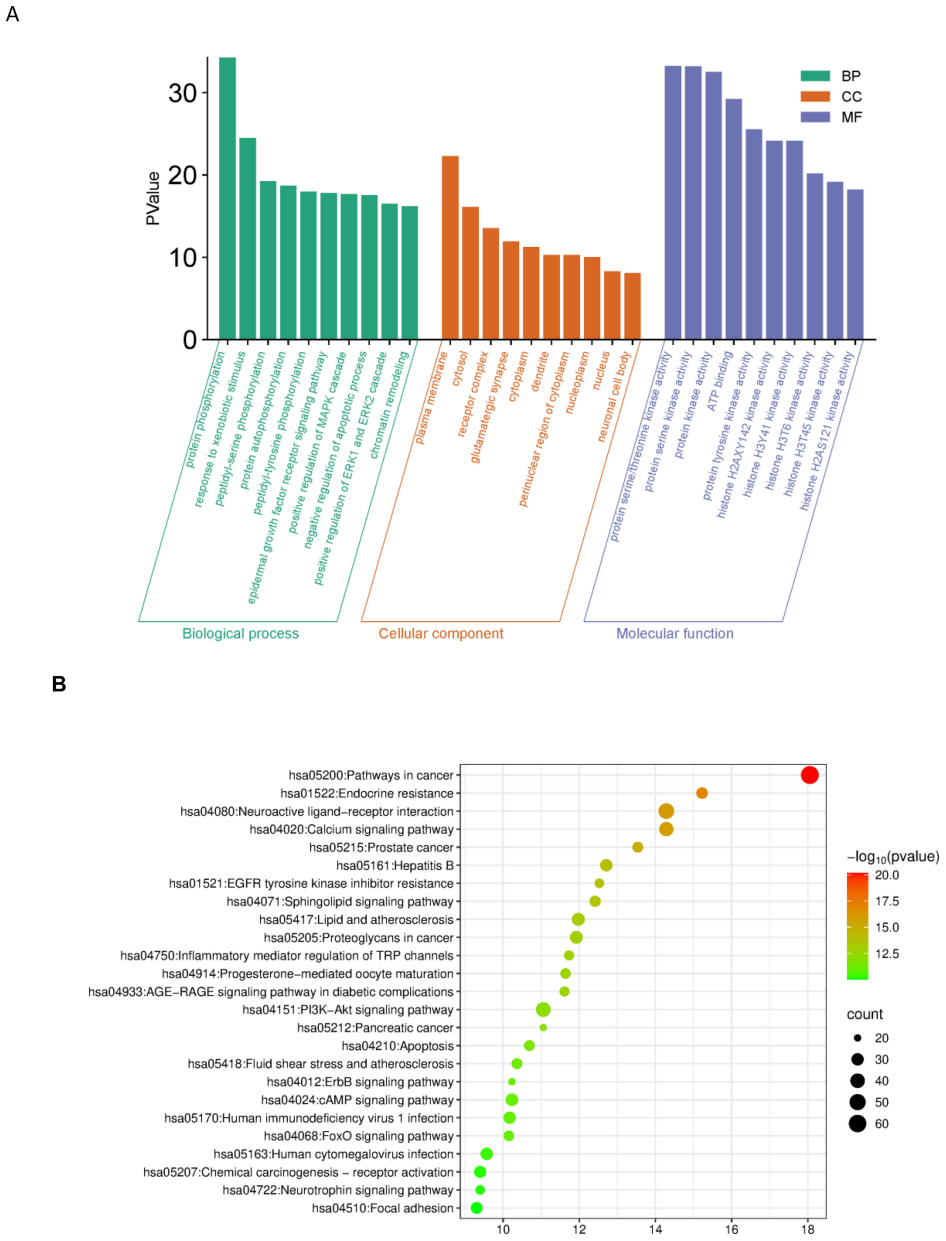
**(A)** GO enrichment analysis (BP/CC/MF). GO (BP/CC/MF) enrichment analysis of potential anti-bladder cancer targets for Myristica fragrans *X-axis: Green bars correspond to Biological Process, orange to Cellular Component, and blue to Molecular Function. Y-axis: The height of the bars represents the inversely transformed P-value, with greater height indicating higher statistical significance.* **(B)** G pathway enrichment analysis. X-axis: Enrichment Factor; Y-axis: Pathway Name; Bubble size: Number of target genes; Bubble color: -log10(P-value), with redder colors indicating more significant enrichment.

GO enrichment analysis indicated significant involvement of the predicted targets in biological processes such as protein phosphorylation, xenobiotic response, EGFR signaling, tyrosine phosphorylation, and regulation of apoptosis (**Figure 3A**).

KEGG pathway analysis identified 25 significantly enriched signaling pathways (P < 0.05). The top enriched pathways included cancer pathways (hsa05200), PI3K-Akt signaling pathway (hsa04151), and apoptosis-related pathways, suggesting that nutmeg may exert anti-bladder cancer effects through multi-pathway regulation (**Figure 3B**).

### 2.5 Molecular docking

**Figure 4:**
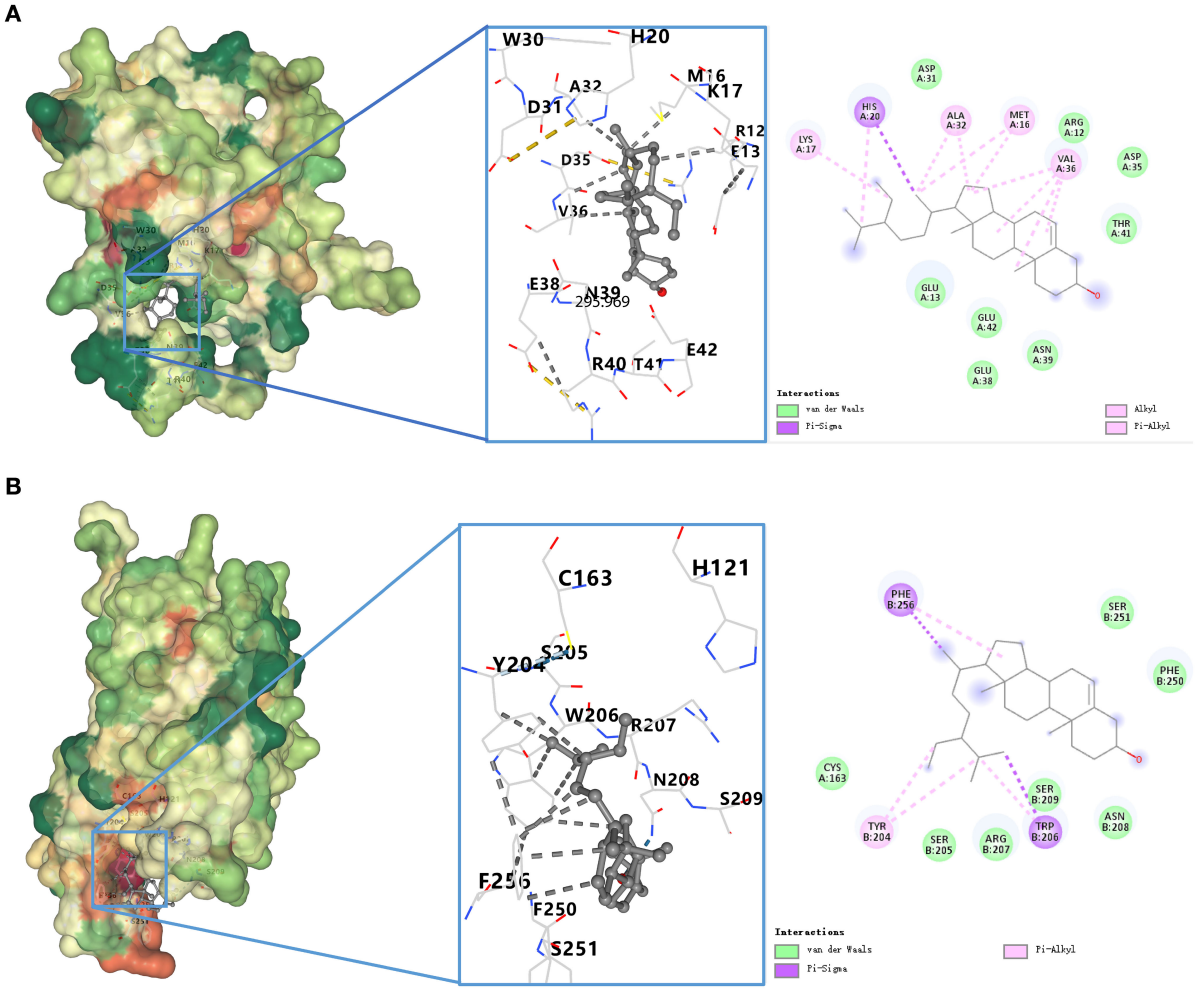
**(A)** Docking model of β-sitosterol with BCL-2. Binding conformation of β-sitosterol with BCL-2 protein (binding energy = -8.6 kcal/mol). **(B)** Docking model of β-sitosterol with CASP3. Binding conformation of β-sitosterol with CASP3 protein (binding energy = -8.3 kcal/mol).

To validate compound–target interactions, molecular docking was performed for eight compounds with available structures against the apoptosis-related targets BCL-2 and CASP3. Among these, β-sitosterol showed the strongest binding affinity (BCL-2: –8.6 kcal/mol; CASP3: –8.3 kcal/mol), surpassing the screening threshold of –5.0 kcal/mol. Docking conformations are shown in **Table 2**, **Figures 4A**, **B**.

**Table 2:** Binding energies of nutmeg compounds with core targets. binding energy of ≤ -5.0 kcal/mol indicates potential binding activity, while ≤ -7.5 kcal/mol suggests strong binding activity.

Active component	Target protein	Binding energy (kcal/mol)
RDK1 (β-谷甾醇)	BCL-2	-8.6
	CASP3	-8.3
RDK2	BCL-2	-6.8
	CASP3	-6.8
RDK3	BCL-2	-6.3
	CASP3	-7.5
RDK4	BCL-2	-7.3
	CASP3	-7.6
RDK5	BCL-2	-7.6
	CASP3	-7.7
RDK6	BCL-2	-7.4
	CASP3	-7.9
RDK7	BCL-2	-7.3
	CASP3	-7.1
RDK8	BCL-2	-7.4
	CASP3	-6.8

Compounds corresponding to RDK numbers are detailed in **Table 1**. BCL-2: B-cell lymphoma 2; CASP3: Caspase-3.

### 2.6 Effects of β-sitosterol on cell viability (IC50 determination)

**Figure 5:**
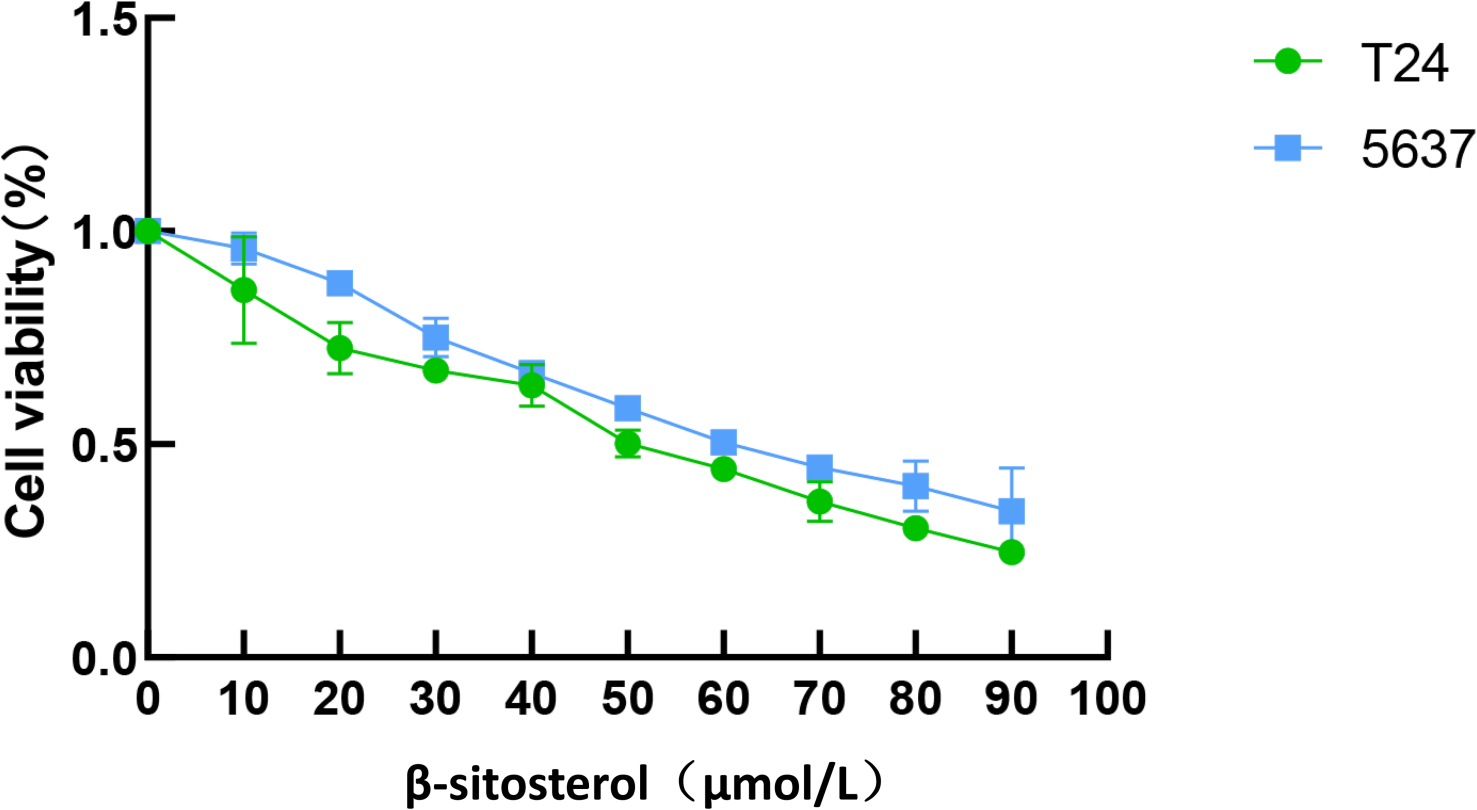
Inhibitory effect of β-sitosterol on bladder cancer cell viability. Effects of β-sitosterol (β-SIT) at different concentrations on the viability of T24 and 5637 cells after 24-hour treatment. Data are presented as mean ± standard deviation (n = 10). *P < 0.05.

Based on network pharmacology and docking results, β-sitosterol (β-SIT) was selected for *in vitro* validation. CCK-8 assays showed that β-sitosterol significantly reduced the viability of T24 and 5637 bladder cancer cells in a concentration-dependent manner after 24 h treatment. The calculated IC50 values were approximately 50 μM in 5637 cells and 60 μM in T24 cells. Concentrations of 40, 50, and 60 μM were used in subsequent experiments (**Figure 5**).

### 2.7 β-sitosterol inhibits cell proliferation

**Figure 6:**
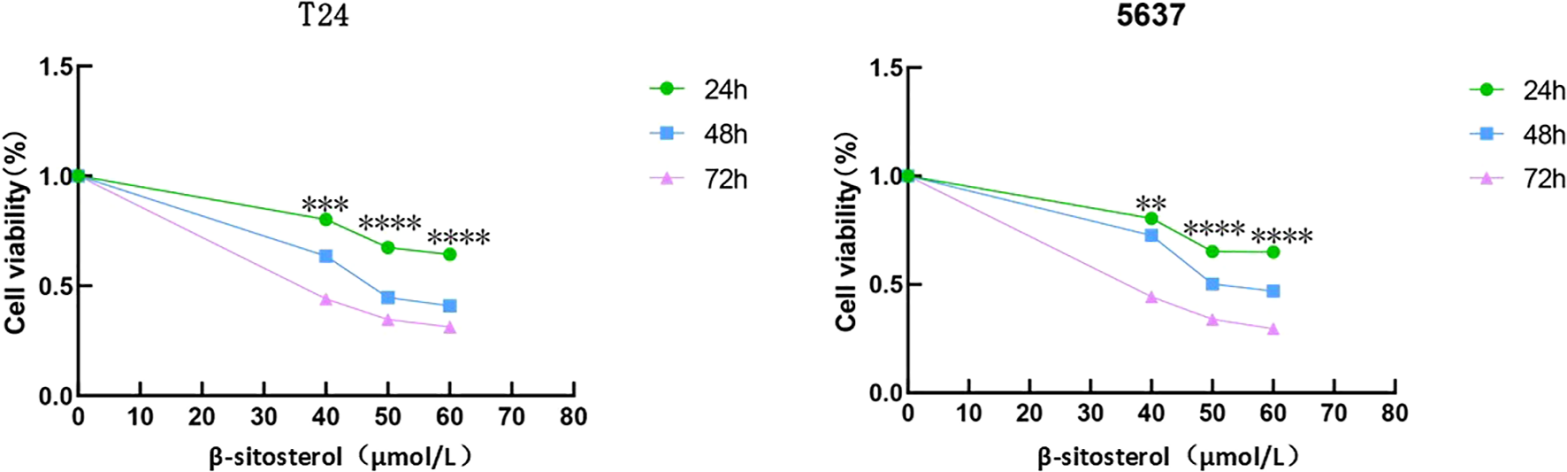
Inhibition of cell proliferation by β-sitosterol. Effects of different concentrations of β-sitosterol (β-SIT) on T24 and 5637 cell proliferation after 24, 48, and 72 hours of treatment. Data are presented as mean ± standard deviation (n = 3). *P < 0.05.

Treatment of T24 and 5637 cells with β-sitosterol for 24, 48, and 72 h significantly reduced proliferation in a time- and dose-dependent manner compared to controls (**Figure 6**).

### 2.8 β-sitosterol suppresses colony formation

**Figure 7:**
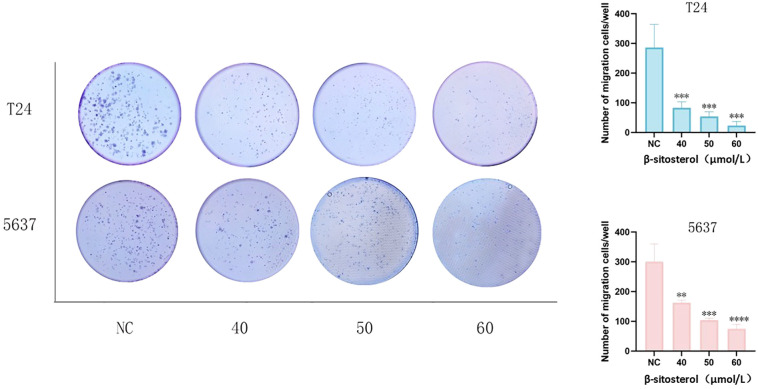
Effects of β-sitosterol on colony formation. Representative images of colony formation in T24 and 5637 cells (crystal violet staining).Quantitative analysis of colony formation in T24 and 5637 cells. Data are presented as mean ± standard deviation (n = 3). *P < 0.05.

Colony formation assays revealed that β-sitosterol markedly reduced the clonogenic ability of bladder cancer cells. Both the number and size of colonies decreased significantly in a concentration-dependent manner (**Figure 7**).

### 2.9 β-sitosterol inhibits cell migration

**Figure 8:**
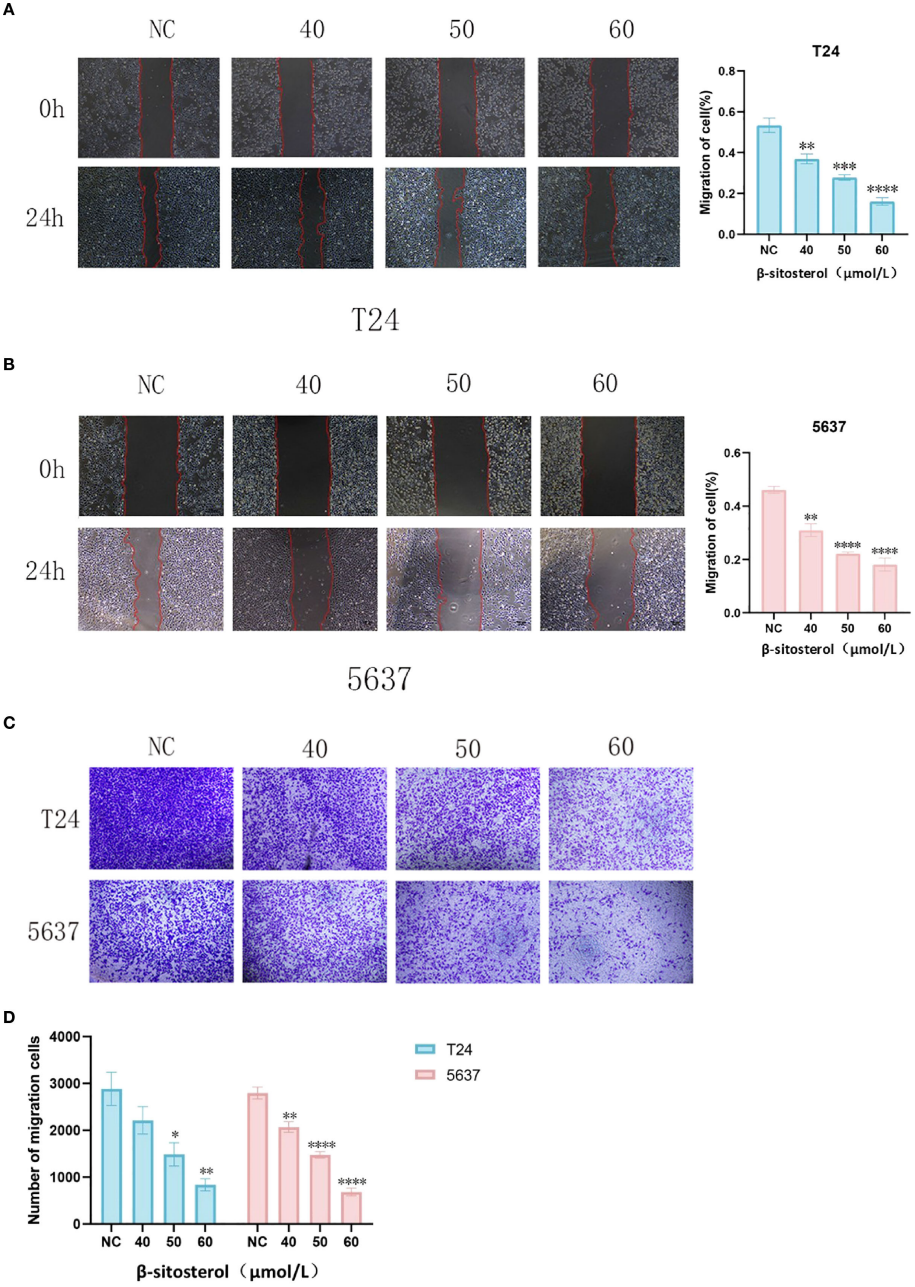
**(A, B)** Wound-healing assay in T24 and 5637 cells. Representative images (40× magnification) and quantitative analysis of wound healing rate in T24 and 5637 cells at 0h and 24h after scratch wound assay. Data are presented as mean ± standard deviation (n = 3). *P < 0.05. (**C)** Representative images of the Transwell migration assay. Representative images of Transwell migration assay (crystal violet staining, 40× magnification). (**D)** Quantitative analysis of migrated cells. Quantitative analysis of migrated cell count in the Transwell assay. Data are presented as mean ± standard deviation (n = 3). *P < 0.05.

Wound-healing and Transwell migration assays demonstrated that β-sitosterol significantly impaired the migratory ability of T24 and 5637 cells (*P* < 0.05). Quantitative analysis confirmed a significant reduction in scratch closure rate and Transwell migration compared to controls (**Figures 8A–D**).

### 2.10 β-sitosterol induces apoptosis

**Figure 9:**
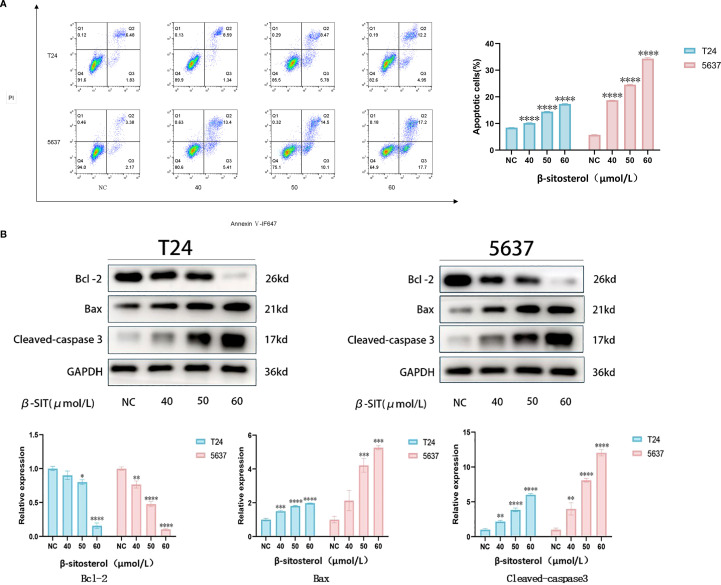
**(A)** Apoptosis detection by flow cytometry. Representative scatter plots of Annexin V-FITC/PI double staining and quantitative analysis of apoptosis rate. Data are presented as mean ± standard deviation (n = 3). *P < 0.05. **(B)** Expression of apoptosis-related proteins by Western blot. Representative bands of BCL-2, BAX, and Cleaved Caspase-3, with relative quantitative analysis of their expression levels normalized to GAPDH. Data are presented as mean ± standard deviation (n = 3). *P < 0.05.

Flow cytometry revealed a dose-dependent increase in apoptosis after 24 h of β-sitosterol treatment in both T24 and 5637 cells (**Figure 9A**). Western blot analysis showed upregulation of Bax and cleaved Caspase-3 and downregulation of BCL-2, indicating that β-sitosterol promotes apoptosis via the BCL-2/BAX/Caspase-3 signaling axis (**Figure 9B**).

## 4 Discussion

Significant advances have been made in elucidating the molecular mechanisms underlying the initiation and progression of bladder cancer, providing a solid theoretical basis for the development of novel therapeutic strategies. Mounting evidence indicates that tumor cell evasion of programmed cell death (apoptosis) is a hallmark of malignancy and a major contributor to therapeutic resistance (15). Thus, dissecting apoptosis-related signaling pathways in bladder cancer is crucial for identifying innovative therapeutic targets with potential clinical value.

Current oncological research predominantly focuses on targeting signaling cascades (16). However, due to tumor heterogeneity and extensive cross-talk among pathways (17, 18), monotherapies often fail to block compensatory activation mechanisms. For instance, in BRAF-mutant melanoma, inhibition of the MAPK pathway induces tumor cells to activate alternative survival pathways via epigenetic or non-genetic mechanisms, leading to treatment escape (19). Consequently, therapeutic efficacy is often limited by drug resistance. To address this complexity, multi-targeted and combination strategies have been increasingly emphasized. For example, in breast cancer, curcumin enhances the efficacy of chemotherapy and mitigates resistance through its multi-targeted effects (20). Hence, the conventional “one disease–one target” paradigm is insufficient for complex tumors such as bladder cancer (21–23).

Network pharmacology, an emerging discipline integrating systems biology, computational analysis, and pharmacology, provides a powerful tool for elucidating the holistic mechanisms of traditional Chinese medicine (TCM). Unlike the linear “one drug–one target” paradigm, network pharmacology emphasizes the “multi-component–multi-target–multi-pathway” model, aligning with the therapeutic principles of TCM (24). This approach has proven particularly effective in unraveling the pharmacological basis of herbal medicines and their synergistic effects in complex diseases (25–27). For instance, studies using network pharmacology and molecular docking revealed that Prunella vulgaris exerts anti-thyroid cancer activity by modulating oxidative stress and immune-related pathways (28).

In recent years, network pharmacology has been widely applied to mechanism studies, drug repurposing, and novel drug design, thereby enhancing the global recognition of TCM and contributing to next-generation drug development paradigms (29). Nevertheless, chemotherapy-induced drug resistance and severe adverse reactions remain major limitations of current treatment strategies, while targeted and immunotherapies are still challenged by high recurrence and tolerance rates in bladder cancer (30). Thus, there is an urgent need for novel agents with low toxicity and high efficacy. TCM extracts, characterized by multi-target synergism, immunomodulatory activity, and low risk of resistance, represent a promising direction in anticancer research. Indeed, several TCM-derived formulations, such as Santalol Injection (Curcuma wenyujin), Kanglaite Injection (Coix seed extract), Huachansu Injection (from toad extract), and Yadanzi Oral Liquid, have already demonstrated clinical efficacy (31–33). Beyond these, new bioactive compounds continue to be identified, and their mechanisms are extensively investigated.

In this context, we employed network pharmacology to identify β-sitosterol (β-SIT), a phytosterol from nutmeg (Myristica fragrans), as a candidate active compound for experimental validation. Nutmeg, a widely used medicinal and dietary substance, has traditionally been employed to warm the spleen and stomach, regulate qi, and alleviate diarrhea (34, 35). Modern phytochemical analyses have revealed its diverse bioactive constituents, including lignans, volatile oils, phenylpropanoids, organic acids, and steroids. Among these, β-sitosterol is of particular interest due to its structural and pharmacological properties.

Our experimental results demonstrated that β-sitosterol significantly inhibited the proliferation and migration of bladder cancer cell lines T24 and 5637 (**Figures 6**–**8**), while promoting apoptosis (**Figure 9A**). Western blot analysis further confirmed that β-sitosterol upregulated pro-apoptotic proteins Bax and cleaved Caspase-3, while downregulating the anti-apoptotic protein BCL-2 (**Figure 9B**). These findings are consistent with molecular docking predictions indicating strong binding affinity between b-sitosterol and BCL-2/CASP3 (**Table 2**). Collectively, these results suggest that β-sitosterol induces apoptosis in bladder cancer cells by modulating the BCL-2/Bax/Caspase-3 signaling axis. Since apoptosis involves coordinated activation of initiator (Caspase-8/9), regulatory (BCL-2 family), and effector (Caspase-3/7) proteins (36–38), the observed regulation of BCL-2 and Caspase-3 highlights the mechanistic significance of β-sitosterol. In addition to apoptosis, our enrichment analysis also highlighted the PI3K-Akt and EGFR signaling pathways as significantly associated with the predicted targets of *Myristica fragrans* in bladder cancer. The PI3K-Akt pathway plays a central role in regulating cell survival, proliferation, and drug resistance in bladder cancer. Aberrant activation of PI3K-Akt signaling has been reported to promote tumor progression and confer chemoresistance, making it an attractive therapeutic target (38). Previous studies demonstrated that phytochemicals, such as curcumin and resveratrol, can modulate PI3K-Akt activity to enhance chemosensitivity in bladder cancer cells (39). Our findings suggest that β-sitosterol may exert similar effects, either directly or indirectly, through the regulation of upstream targets within this pathway. Likewise, the EGFR pathway emerged as another enriched axis in our network pharmacology analysis. EGFR overexpression and mutation are well-documented drivers of bladder cancer progression, contributing to enhanced proliferation, invasion, and epithelial–mesenchymal transition (EMT). Inhibition of EGFR has been shown to suppress tumor growth and improve outcomes in preclinical bladder cancer models (40). Although our current experimental validation primarily focused on apoptosis-related targets, the integration of EGFR-related signaling pathways indicates a broader mechanistic spectrum of β-sitosterol’s action. Future studies should further investigate whether β-sitosterol can modulate EGFR-mediated signaling, either alone or in combination with standard therapies, to achieve synergistic antitumor effects.

In summary, this study combined network pharmacology and *in vitro* validation to elucidate the anti-bladder cancer mechanism of nutmeg and its key constituent β-sitosterol. The results indicate that β-sitosterol suppresses proliferation and migration while inducing apoptosis, mainly through the BCL-2/Bax/Caspase-3 axis, and potentially through additional pathways such as PI3K-Akt, EGFR, and PI3K/mTOR. These findings provide mechanistic evidence supporting nutmeg and β-sitosterol as potential adjuvant therapies for bladder cancer. Beyond bladder cancer, β-sitosterol has also demonstrated anticancer effects in other tumor types, including breast and lung cancers, where it was reported to inhibit proliferation and induce apoptosis (41, 42). Although our study focused on bladder cancer, these findings suggest that β-sitosterol may have broader therapeutic relevance, and future research should evaluate its activity across multiple cancer models to clarify its general applicability.

## 5 Limitations

This study has several limitations. First, the findings rely on *in vitro* assays and network pharmacology, which need validation in animal models and clinical settings. Second, while β-sitosterol was identified as the main compound, other nutmeg constituents with potential anticancer activity (e.g., isoguaiacin, galbacin) were not examined. Third, β-sitosterol shows low oral bioavailability and limited stability *in vivo*, which may restrict its clinical use. In addition, although it is generally considered safe, its effects on organs such as the liver and kidneys remain unclear, and evidence for long-term safety is lacking. Finally, pharmacokinetic and pharmacodynamic studies, together with systematic toxicological assessments, are required to clarify its ADME profile and safety. Future work should also explore possible synergistic effects among nutmeg-derived compounds to fully establish its therapeutic potential.

## 6 Conclusion

This study integrates network pharmacology and experimental validation to provide preliminary evidence that nutmeg exerts anti-bladder cancer effects through multi-component, multi-target, and multi-pathway mechanisms. β-sitosterol, identified as a core bioactive compound, significantly suppresses the proliferation and migration of bladder cancer cells and induces apoptosis by modulating the BCL-2/Bax/Caspase-3 axis. These findings support the potential of nutmeg and β-sitosterol as adjuvant therapeutic candidates for bladder cancer. Future investigations should include in-depth mechanistic studies, *in vivo* validation, and clinical trials to establish efficacy, safety, and therapeutic applicability.

## Data Availability

The original contributions presented in the study are included in the article/supplementary material. Further inquiries can be directed to the corresponding authors.
